# Childhood-Onset Schizophrenia: Insights from Induced Pluripotent Stem Cells

**DOI:** 10.3390/ijms19123829

**Published:** 2018-11-30

**Authors:** Anke Hoffmann, Michael Ziller, Dietmar Spengler

**Affiliations:** Department of Translational Research in Psychiatry, Max Planck Institute of Psychiatry, 80804 Munich, Germany; hoffmann@psych.mpg.de (A.H.); michael_ziller@psych.mpg.de (M.Z.)

**Keywords:** childhood-onset schizophrenia (COS), induced pluripotent stem cell (iPSC), copy number variation (CNV), early neurodevelopment, neuronal differentiation, synapse, dendritic arborization, miRNAs

## Abstract

Childhood-onset schizophrenia (COS) is a rare psychiatric disorder characterized by earlier onset, more severe course, and poorer outcome relative to adult-onset schizophrenia (AOS). Even though, clinical, neuroimaging, and genetic studies support that COS is continuous to AOS. Early neurodevelopmental deviations in COS are thought to be significantly mediated through poorly understood genetic risk factors that may also predispose to long-term outcome. In this review, we discuss findings from induced pluripotent stem cells (iPSCs) that allow the generation of disease-relevant cell types from early brain development. Because iPSCs capture each donor’s genotype, case/control studies can uncover molecular and cellular underpinnings of COS. Indeed, recent studies identified alterations in neural progenitor and neuronal cell function, comprising dendrites, synapses, electrical activity, glutamate signaling, and miRNA expression. Interestingly, transcriptional signatures of iPSC-derived cells from patients with COS showed concordance with postmortem brain samples from SCZ, indicating that changes in vitro may recapitulate changes from the diseased brain. Considering this progress, we discuss also current caveats from the field of iPSC-based disease modeling and how to proceed from basic studies to improved diagnosis and treatment of COS.

## 1. Introduction

Schizophrenia (SCZ) is a highly heritable, devastating mental disorder with a lifetime prevalence of ≈1% worldwide [1]. First episode psychosis typically manifest in early adulthood followed by recurrent episodes that frequently give way to a chronic course that confers substantial mortality and morbidity. As of yet, no cure is available and life expectancy of patients with SCZ is reduced by 15 to 30 years [2,3]. Around 4% of the patients experience early-onset schizophrenia (EOS) either during childhood prior to the 13th birthday (i.e., COS) or during adolescence up to the age of 17 years and carry a particular worse diagnosis [4].

Numerous hypothesis, observational, and experimental, have been put forward to explain the etiology and pathogenesis of SCZ with no consensus established so far [5]. Among these, the much-noticed neurodevelopmental hypothesis of SCZ posits that deviations in early brain development predispose to later vulnerability when critical processes of normal maturation call into operation damaged structures. Similar to other fields of early-onset disease, the study of patients with COS showed that early SCZ is characterized by increased symptom severity and a higher genetic load. This indicates a greater genetic salience for neurodevelopmental deviations and suggests that studying COS can also advance insight into disease-traits that develop more subtly in an adult-onset patient group [6]. Early-onset of disease reduces also the contribution of confounding environmental factors across life course and enables a less-clouded sight on the actual biology underpinning SCZ.

Due to the impossibility to isolate brain tissue from living patients, and the limitations of postmortem studies (scarcity of tissue availability, confounding effects from treatments, aging, and life history) patient-specific iPSCs offer a unique opportunity to study living human neuronal cells. iPSCs capture a donor’s genotype including disease related genetic risk factors, known and unknown, and can be differentiated in virtually any cell type including early neural cells of potential relevance to COS. Differences in early cellular and molecular endophenotypes from case/control studies can inform on perturbations in neurodevelopmental pathways and on potential deviations in patients with COS. Patient-specific iPSC studies on COS are of particular interest given that early perturbations in vitro may couple more directly to early than later pathology and thus offer a better handle on cause–effect relationships.

Here, we will examine this hypothesis by considering the clinical picture and course of COS, and recent insights into the genetics of AOS and COS. Against this background, we discuss how iPSC-derived neuronal cells from early developmental stages differ between carriers of high-risk structural variations variants for COS or patients with COS vs. healthy donors. We further ask whether these molecular and cellular alterations do bridge to early brain development. Concluding, we address present caveats in patient-specific disease modeling and upcoming improvements from the field.

The literature selection process for this review was conducted in the databank PubMed via combinations of the search terms “schizophreni*”, “childhood”, “early-onset”, “induced pluripotent stem cell*”, “genetic*”, and “psychosis” with date limits from 2007 (first report on iPSCs [7]) to September 2018. Additional searches included scrutiny of similar articles suggested by PubMed, of references from the identified publications, and of citatory publications identified by Google Scholar^®^.

## 2. The Neurodevelopmental Hypothesis of COS

COS is a rare disorder affecting 1 in 10,000–30,000 children [8]. Prior to the 20th century, bizarre behavior, social withdrawal, catatonia, and/or psychosis in children were regarded as undifferentiated conditions, labelled as “hereditary insanity”, “dementia praecox”, or “developmental idiocy” [9]. Today’s diagnostic criteria are the same as in AOS and concern multiple domains of behavior and cognition with a prominent role of psychotic symptoms. Hallucinations, delusions, and disorganized thinking [10,11] frequently concur with impairments in social communication, as well as in motor, volitional, and emotional abnormalities [12]. Longitudinal studies have corroborated that diagnostic stability is high in EOS at around 80–90% [13,14]. Outcome of patients over 40 years with EOS is consistently worse relative to AOS [14,15,16] with the worst clinical and psychosocial outcomes in COS [17]. EOS manifests greater neurodevelopmental deviance early in life, yet it is clinically and neurobiological continuous with AOS [12,18,19].

The discovery of first-generation antipsychotics in the 1950s, known as typical antipsychotics [20], has transformed the treatment of SCZ. Although the first atypical antipsychotic, clozapine, was discovered in the 1960s and introduced clinically in the 1970s, most second-generation drugs, known as atypical antipsychotics, have been developed more recently. Both generations of medication are thought to block receptors in the brain’s dopamine pathways with atypicals acting on serotonin receptors additionally. Recent data suggest a greater efficacy of clozapine, relative to other antipsychotics, in COS than in AOS [21] and raise the perspective that COS could offer a unique opportunity to learn to what degree neurodevelopmental deviations in SCZ could respond to current pharmacotherapy.

**Figure 1:**
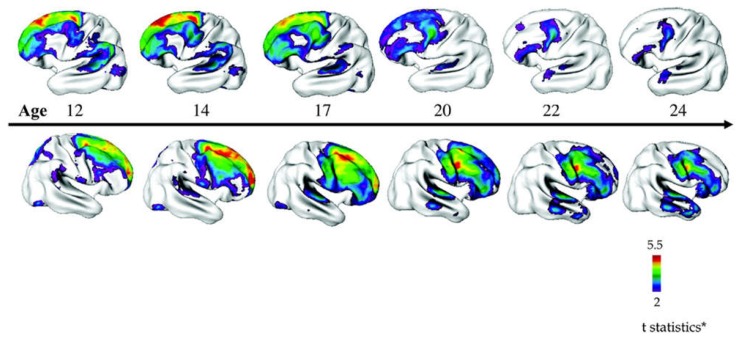
Progression of cortical gray matter loss in patients with childhood-onset schizophrenia (COS) (*n* = 70) relative to age-, sex-, and scan interval-matched healthy individuals (*n* = 72). Brain templates illustrate areas of significant thinning in patients with COS in a ‘front-to-back’ pattern from adolescence to young adulthood (age 12–24 years). Side bar shows *t* statistic with threshold to control for multiple comparisons. Figure 1 is reproduced from Gogtay [26] by permission of Oxford University Press, adapted by Greenstein et al. [27] by permission of John Wiley and Sons.

At the macroscopic scale, early structural MRI (magnetic resonance imaging) studies by the National Institute of Mental Health (NIMH) suggested a pattern of reduced cerebral volumes and larger ventricles in COS consistent with findings from AOS [22]. Longitudinal follow-up studies further showed that typically developing children undergo a small decrease in cortical gray matter (≈2%) in the frontal and parietal regions throughout adolescence (Figure 1). By contrast, children with a history of COS experience exaggerated gray matter losses (≈8%) in frontal, parietal, and temporal lobes [22]. These losses originated in the parietal lobes and spread anteriorly over time until they leveled off in early adulthood when SCZ typically manifests [23]. This pattern fits well the hypothesis of an exaggerated synaptic pruning during critical neurodevelopmental time windows in SCZ [23,24] and supports that COS evolves from a vulnerable brain (Figure 1). It is also worth mentioning that these changes were specific for COS and were not shared with other age- and gender-matched patients with psychotic symptoms diagnosed as multidimensional impaired [25]. In addition, children with COS display losses in global gray matter and cortical thickness in childhood that with age approach those detected in AOS.

Interestingly, non-affected siblings of patients with COS show equally a pattern of decreased thickness in the frontal temporal and parietal lobes during childhood and adolescence that normalizes in early adulthood. This indicates that genetic risk factors underpinning COS interact in a complex manner with the environment leading to overt psychopathology or normalization of risk phenotypes [26,28]. Beyond structural changes, functional MRI studies on patients with COS suggest exaggerated long-range connectivity implicating greater global connectedness and efficiency. Concomitantly, short-range connectivity is impaired in patients with COS implicating disrupted modularity [29,30]. Similar structural deviations have been detected in neonates at high risk for SCZ re-enforcing that COS is contiguous to SCZ [31].

These neuroimaging studies raise the question how macroscopic findings can be explained microscopically. In the absence of neurodegenerative lesions and gliosis, histopathological studies have scrutinized the cytoarchitecture of the cerebral cortex for changes in the size, location, distribution, and packing density of neurons and their synaptic connections. Three putative alterations have caught particular attention: abnormal neuronal organization (dysplasia) in lamina II (pre-alpha cells) and lamina III of the entorhinal cortex [32], disarray of hippocampal neurons [33], and an altered distribution of neurons in the subcortical white matter [34]. These findings seemed to implicate impairments in neuronal migration and cytoarchitecture and were taken as strong evidence for the neurodevelopmental hypothesis of SCZ. Disappointingly, none of these findings has been firmly recapitulated so far. However, a bulk of histopathological studies collaborate the presence of smaller cortical and hippocampal pyramidal neurons, decreased cortical and hippocampal synaptic markers, and decreased dendritic spines as cardinal symptoms in AOS [35]. In light of our limited understanding of SCZ’s neuropathology, future studies are needed to resolve the dynamic nature of the disorder. A one-fits-all model is unlikely to reflect the complex nature of changes in development, adult plasticity, and aging. In fact, the diversity of postmortem cellular pathology may conform to accruing evidence for multi-factorial genetic heritability in SCZ.

## 3. The Genetic Architecture of AOS and COS

Heritability for AOS is about 60% and 80% in national family [36,37] and twin studies [38,39]. Similarly, twin studies on patients with COS indicate a heritability about 88% [40]. Additionally, family studies on patients with COS show an increased rate of schizophrenic spectrum disorders pointing to familial transmission [41].

Genome-wide association studies (GWAS) have identified many common genetic variants (mostly single nucleotide polymorphism, SNPs) of small effect size that explain between one-third and one-half of the genetic variance [42]. A seminal meta-analysis on 36,989 patients with AOS and 113,075 controls discovered 128 common variant associations encompassing 108 independent loci that met the criterion of genome-wide statistical significance (e.g., 5 × 10^−8^) [43]. These loci covered multiple regions enriched in genes regulating glutamatergic, calcium, and G-protein coupled receptor signaling, neuronal ion channels, synaptic function and plasticity, and several neurodevelopmental regulators. A subsequent GWAS study has replicated 93 of these risk loci and identified additionally 52 new loci associated with AOS [44]. Most recently, a GWAS study for shared risk across major psychiatric disorders (including AOS) has highlighted fetal neurodevelopment as a key mediator of vulnerability: four genome-wide significant loci encompassed variants thought to regulate genes expressed in radial glia cells and interneurons in the developing cortex during midgestation [45].

Despite these advances, it is important to realize that risk-associated SNPs typically map to non-coding genomic regions equally represented by intergenic and intronic regions [46]. These SNPs are not necessarily the causal genetic variant underlying the association nor do they identify the causative gene(s). Future studies still have to identify those SNPs that encode a regulatory function and contribute causally to SCZ [47].

Over the last few years, an increasing number of copy number variations (CNVs) has been shown to increase the risk for SCZ. CNVs are typically caused by the presence of region-specific, repetitive DNA sequences, termed low copy repeats (LCRs). Recombination between adjacent and homologous LCRs via non-allelic homologous recombination (NAHR) results in deletions or duplications of the DNA stretches between the repeats. These CNVs tend to recur at the same chromosomal positions flanked by the LCRs, while other mechanism such as non-homologous end joining (NHEJ) can cause non-recurrent CNVs that contain different breakpoints. In any case, most of these de novo mutations, recurrent and non-recurrent, are likely to reduce fecundity and are therefore rarely transmitted [48].

With the advent of microarrays, it became feasible to interrogate the whole genomes of large case/control cohorts for the presence of CNVs that enhance the risk for SCZ. These risk CNVs comprise deletions at 1q21.1, 2p16.3 (contains only *Neurexin 1* with a role in neurotransmission and synaptic contact formation), 3q29, 15q11.2, 15q13.3, and 22q11.2, and duplications at 1q21.1, 7q11.23, 15q11.2-q13.1, 16p13.1, and proximal 16p11.2. A recent combined meta-analysis of 21,094 patients with SCZ and 20,227 controls has shown in a small fraction (1.4%) of the cases genome-wide significant association with CNVs and has confirmed the role of most previously implicated CNVs including 1q21.1, 2p16.3, 3q29, 7q11.2, 15q13.3, distal and proximal 16p11.2 and 22q11.2 (Table 1) [49]. Furthermore, the researchers identified another eight loci that showed suggestive evidence of association with SCZ (Table 1).

The aggregate CNV burden was enriched for genes controlling synaptic function (OR = 1.68, P = 2.8 × 10^−11^) and neurobehavior (in mice). Carrying a CNV risk allele explains only 0.85% of the variance in SCZ liability relative to 3.4% by the 108 genome-wide significant loci [43]. However, risk CNVs show significantly greater effects on SCZ risk (Table 1) than common SNP variants (OR < 1.3). It is worth noting that these risk CNVs associate also with a distinct spectrum of disorders (autism spectrum disorder (ASD), developmental delay, and congenital malformation) indicating that deviations in early neurodevelopment are shared across these disorders [48].

An early study on CNVs on patients with AOS, COS, and ancestry-matched controls found that 15% of patient with AOS had novel structural variants compared with 5% of controls [50]. By contrast, 20% of patients with onset of SCZ before 18 years of age and 28% of patients with COS carried one or more rare structural variants. Structural variations in patients with SCZ were enriched in genes controlling brain development, especially those involving neuregulin and glutamate pathways. In support of this finding, the NIMH COS study showed that 10% of the patients with COS exhibited large chromosomal abnormalities at rates significant higher than those measured in the general population or in patients with AOS [51]. This finding has been collaborated in a follow-up study [6]: a total of 11.9% of patients with COS harbored at least one CNV and 26.7% had two. Among these, 4% showed a 2.5–3 Mb deletion mapping to 22q11.2, a rate higher than that reported for AOS (0.3–1%) or the general population (0.2%), and the highest rate reported for any clinical population to date. Patients with COS also carried additional genomic lesions at 8q11.2, 10q22.3, 16p11.2, and 17q21.3 that had been previously associated with intellectual disability or autism supporting the pleiotropic role of these CNVs in early brain development.

Beyond CNVs, polygenetic risk scores derived from selected common risk variants for SCZ predict effectively COS status: patients with COS had higher genetic risk scores for SCZ (and autism) than their siblings suggesting that patients with COS have more salient genetic risk than do patients with AOS [43].

Taken together, COS is a rare form of SCZ in which both common variants of small effect (SNP) and rare variants (CNV) of large effect conspire together. Common and rare risk variants are more frequent in patients with COS than in patients with AOS. At the same time, patients with COS share rare variants associated with ASD. In essence, patients with COS carry a particular high risk for SCZ that underpins earlier manifestation and a more severe course relative to AOS. Regarding the neurodevelopmental hypothesis of SCZ, patients with COS are therefore expected to manifest more salient neurodevelopmental deviations than patients with AOS. With this in mind, future studies are mandatory to link common and rare risk variants-to genes-to function in order to understand the biology underlying COS and to develop better treatments. To approach this daunting task, iPSC-based studies can provide an important tool to study the regulatory effects of genetic variants, candidate genes, and the overall effect of these variants and their interconnected networks [52], known and unknown, on cellular and molecular endophenotypes in disease-relevant human cells.

## 4. iPSCs Provide Unique Access to Early Neurodevelopment in AOS and COS

**Figure 2:**
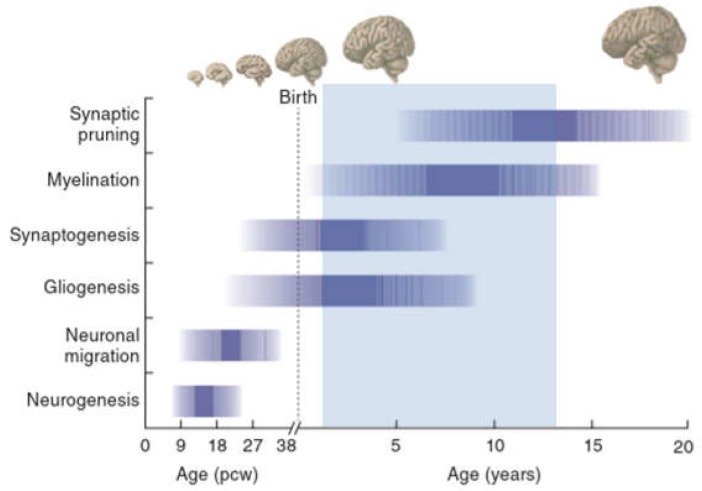
A timeline of human development during prenatal (in postconception weeks, pcw) and postnatal (in years) periods. The shaded horizontal bars represent the approximate timing of key neurobiological processes and developmental milestones. The light-blue overlay marks the period during which childhood-onset schizophrenia (COS) typically manifests. Gross anatomical features and the relative size of the brain at different stages are illustrated at the top. Adapted by Springer Nature (https://www.nature.com/nature/), Developmental timing and critical windows for the treatment of psychiatric disorders, Oscar Marín, 2016 [53].

Human brain development starts with the differentiation of neuronal progenitor cells (NPCs) in the third gestational week and subsists through at least late adolescence (Figure 2). Neural tube formation, neural patterning, and NPC differentiation take place in embryonic and early fetal periods and proceed to neuron production, migration, and differentiation in later fetal and early postnatal periods.

Regressive and progressive neuronal processes, remodeling of synaptic contacts and circuitries, and myelination evolve postnatally and subsist beyond adolescence [54,55,56]. Cortical circuits are refined through pruning of excitatory synapses, proliferation of inhibitory circuits, and remodeling of pyramidal dendrites in early adulthood [57,58]. These modulatory processes serve to fine-tune excitatory–inhibitory cortical balance and appear perturbed in patients with SCZ.

Genetic studies on AOS and COS suggest combinatorial contributions of many variants across a host of loci, rather than one or a few penetrant single-gene mutations. These highly polygenic states cannot be engineered into animal models, as they demand replicating large portions, if not the entirety, of the human genome. Hence, human models are urgently needed to decode polygenic contribution to disease initiation and manifestation. Human iPSCs retain the unique genetic signature of the donor and provide insight into the relationship between the donor’s genotype and an in vitro endophenotype. By now, human iPSCs are routinely generated from skin biopsies or peripheral blood mononuclear cells [59,60]. iPSCs can be differentiated in disease-relevant neurons and astroglia in order to re-enact altered trajectories of brain development in a diseased individual. In distinction to postmortem brain tissue, human iPSCs are not confounded by secondary disease processes, therapy, or life history. Therefore, iPSC studies are particular promising for the analysis of the effects of polygenic risk on programs underpinning cellular and molecular endophenotypes in the developing and early postnatal brain.

Encouragingly, comprehensive RNA expression profiling of human brain tissues from early embryonic to late adult postmortem stages has shown that neuronal cells produced from iPSCs closely recapitulate the progression from early embryogenesis to late fetal periods in vitro and yield neuronal cells of various stages of maturity [61,62,63,64,65,66]. Immature neurons and networks express molecules and processes that are not operative in the adult and follow a crucial developmental sequence that is instrumental in the formation of functional entities. While caution needs to be exercised to extrapolate from iPSC-derived cell stages to those in adolescents and adults, they provide unique access to explore molecular and cellular endophenotypes and cause–effect relationships in living disease-relevant cell types from early neurodevelopmental stages from patients with COS.

## 5. Tracing Early Neurodevelopment in Patients with COS

In 2011, Brennand and coworkers firstly reported the generation of iPSC-derived neuronal cells from patients with familial SCZ and detected significant reductions in neuronal connectivity, neurite outgrowth, and dendrite formation in forebrain neurons from patients relative to controls [67]. This influential work has prompted an increasing number of patient-specific iPSC studies on AOS [68], but also on bipolar disease [69]. Here, we consider iPSC-based case/control studies on carriers of high-risk structural variations associated with COS or on patients diagnosed COS vs. healthy controls. For clarity, experimental approaches, and key findings are summarized in a tabular format.

### 5.1. Role of the 22q11.2 Microdeletion as Risk Factor for AOS and COS

The 22q11.2 deletion syndrome (22q11.2.DS), also known as DiGeorge or velocardiofacial syndrome, has an incidence of 1 in 2000–4000 live births [70]. Typical microdeletions are either 3 Mb in size (covering about 60 known genes) or 1.5 Mb in size (covering about 35 known genes). Most of the genes inside these regions are expressed in the brain. The severity of the disorder is unrelated to the size of the deletions indicating that genes residing within the 1.5 Mb region are critical to the etiology of the syndrome. Frequent physical manifestations consist of craniofacial and cardiovascular anomalies and immunodeficiency among others symptoms. Patients with 22q11.2.DS also show cognitive and behavioral impairments and a high risk for ASD, neurodevelopmental delay, and SCZ [48]. In fact, the identification of rare and highly penetrant de novo structural variations at 22q11.2 in sporadic cases of SCZ provided the first evidence for the role of rare recurrent mutations in SCZ susceptibly [71]. This structural mutation is detected in up to 1% and 4% of AOS and COS cases, respectively [6] and up to one-third of all patients with 22q11.2.DS develop SCZ or schizoaffective disorder (SAD). Noteworthy, there are no major clinical differences in core psychopathology, treatment response, neurocognitive profile, and imaging anomalies between schizophrenic patients with 22q11.2.DS or an intact chromosome 22 [72]. In fact, many patients with 22q11.2.DS show no serious intellectual disability and congenital abnormalities can be so subtle that they appear undistinguishable from other patients with SCZ. Consistent with these findings, intellectual ability and length of the microdeletion do not appear to be major risk factors for SCZ associated with the 22q11.2 microdeletion.

In 2011, Pedrosa et al. [73] firstly reprogrammed fibroblasts (Table 2) from a patient with AOS carrying a 22q11.2 microdeletion, a high risk factor for COS, and two healthy controls. Two iPSC lines from patients with SCZ, one with adult-onset SCZ and one with COS (Table 3) were obtained additionally from Brennand et al. [67]. iPSC quality control (Table 2) consisted of immunocytochemistry (ICC), teratoma (Tera) and embryoid body formation (EB), and karyotype analysis (G-B, G-banding; FISH, fluorescence in-situ hybridization).

Neural induction involved embryoid body (EB) and neural rosette formation (an in vitro equivalent to the neural tube) (Table 4). Subsequently, NPCs were manually dissected and differentiated in mixed cultures of forebrain glutamatergic neurons that were able to fire action potentials after two months in culture. Expression profiling across undifferentiated and differentiated case/control iPSCs showed no gross differences except for the pluripotency markers OCT4 and NANOG. These markers declined more slowly during glutamatergic differentiation of the iPSCs derived from the patient with AOS and a 22q11.2 microdeletion relative to the other samples.

Analysis of homogenized cultures from iPSC-based case/control studies can disguise the detection of disease-relevant signals due to the high heterogeneity of cell types, broadly varying maturation states, and of differences in differentiation capacity (see also Section 5.5 and Section 6). In a follow-up study, Belinsky et al. [74] sought to address this concern by combining patch recording with single-cell PCR (polymerase chain reaction) for expression profiling of a selected panel of genes from neurodevelopment, GABAergic and glutamatergic signaling among others. Neurons derived from a patient with adult-onset SCZ carrying a 22q11.2 microdeletion and one control (Table 3) showed similar active and passive electrical activities across the entire time course of neuronal differentiation. At the same time, electrical activities were poorly synchronized due to varying maturation states. However, once patient-derived neurons developed electrical activities, the expression of genes relevant for GABAergic, glutamatergic, and dopaminergic specification appeared subtly deregulated relative to the control.

The development of complex behaviors and higher cognitive functions in human involves the development of highly specialized cell types and circuitries that may be impaired in psychiatric disorders such as COS/AOS. Accruing evidence suggests that genomic DNA in the brain contains characteristic somatic genetic variations relative to non-brain tissues [87]. These variations comprise mutations, chromosomal aneuploidy, or microdeletions, and the dynamics of non-long terminal repeat (LTR) retrotransposons. All of these variations contribute potentially to the production of functionally diversified brain cells. Among the known retrotransposons, only long interspersed nucleotide element-1 (L1) possesses autonomous retrotransposition activity that is required for the insertion of new L1 copies. L1 shows retrotransposition activity in rat hippocampal NPCs [88], human embryonic stem cells, and human fetal and adult brain [89]. Furthermore, increased L1 retrotransposition was detected in a mouse model of Rett syndrome and in Rett patients, suggesting a role in neurodevelopmental disorders [90].

Considering these findings, Bundo et al. [75] investigated L1 activity in postmortem prefrontal cortex from patients with AOS and iPSC-derived neurons from two patient with AOS carrying 22q11.2 microdeletion, a known high risk factor for COS. Whole-genome sequencing showed that brain-specific L1 insertion in patients with AOS localized preferentially to genes involved in synapse formation and function, cell adhesion, and cytoskeleton among other processes relevant to SCZ [75]. L1 copy number was unrelated to confounding factors (e.g., age, age of onset, and duration of illness) and emerged from early neurodevelopmental stages, at least in the prefrontal cortex (Table 5).

Interestingly, L1 insertion was also increased in iPSC-derived neurons containing the 22q11.2 microdeletion relative to controls supporting the role of this variation as risk factor for SCZ. Remember that iPSC-derived cells match early embryonic to early postnatal stages and thus provide in vitro evidence for a role of L1 retrotransposition during early neurodevelopment. In support of this hypothesis, immune activation by poly-I:C treatment of rat dams (a translational model for the generation of schizophrenia-like symptoms in the offspring) led to an increase of L1 copy number in the brain. Hence, an increase in L1 insertion in response to environmental or genetic risk factors may increase the vulnerability for SCZ by impairing synaptic and related functions in neurons, rather than representing a primary cause of the disease.

Beyond mRNAs, the developing human brain expresses also high levels of microRNAs (miRNAs) that regulate neural lineage and cell fate decisions, differentiation, and neuronal maturation [91,92]. miRNAs are noncoding RNAs of ~70 nucleotides in size (pri-miRNAs) that are cleaved by a nuclear protein complex encompassing DGCR8 and DROSHA into precursor RNAs (pre-miRNAs) [93]. Latter are further cleaved by DICER to yield single stranded ~22 nucleotide mature miRNAs that are incorporated into the RNA induced silencing complex (RISC). Subsequently, miRNAs target through a 6- to 8-base pair complementary ‘seed region’ one or more mRNAs with each miRNA potentially downregulating up to hundreds of downstream targets [93]. Changes in miRNA expression profiles have been detected in SCZ, autism, and major depressive disorder (MDD) [94]. For example, miRNA-137 (miR-137) maps to a risk locus of SCZ [95,96] and seems to downregulate disease related genes like *TCF4* (transcription factor 4) or *CACNA1C* (calcium channel, voltage-dependent, L-type, alpha-1C subunit) [97,98].

Noteworthy, *DGCR8* (DiGeorge syndrome critical region gene 8) resides inside the 22q11.2 microdeletion. Reduced expression of *DGCR8* slows the conversion of a subset of pri-miRNAs to pre-miRNAs and results in a dampened production of a particular subset of mature miRNAs [99]. Additionally, the 22q11.2 region harbors *MIR-185* that targets other candidate genes relevant to SCZ, to hippocampal dendritic spine density, and to synapse function [100].

Given these premises, Zhao et al. [76] sought to analyze the miRNA profiles in living neurons generated from patients with (i) SAD or AOS carrying the 22q11.2 microdeletion, a high risk factor for COS, or (ii) with COS carrying an intact chromosome 22 (Table 3). MiRNA sequencing of day 14 neurons (Table 5) from six controls (with multiple clones for two controls) and from six patients with SAD, AOS, or COS, detected 45 differentially expressed miRNAs (13 lower in SCZ; 32 higher). Among these miRNAs, six were significantly downregulated in neurons carrying the 22q11.2 microdeletion, including four miRNAs that map to the 22q11.2 microdeletion (miR-1306-3p, miR-1286, miR-1306-5p, and miR-185-5p), and two that did not (miR-3175 and miR-3158-3p). This result suggests that some miRNAs are downregulated independently of *DGCR8* possibly by one or more of the transcriptional and chromatin regulators that map to this chromosomal region. In support of this finding, 32 differentially expressed miRNAs were upregulated in the 22q11.2 microdeletion samples, rather than downregulated. Functional pathway analysis of the differentially expressed miRNAs showed enrichment for genes relevant to neurological and psychiatric disorders and neurodevelopment. For example, miR-34c, a member of the miR-34 family, is predicted to target *CNTNAP1*, *CNTNAP2*, *GABRA3*, *RELN*, *FOXP2*, *NRXN2*, and ANK3, while mi-R34a plays a role in neural stem cell (NSC) differentiation. Moreover, many of the differentially expressed miRNAs in iPSC-derived neurons carrying a 22q11.2 microdeletion were shared with clinical/autopsy samples drawn from the general population of AOS and ASD indicating that the underpinning molecular genetic networks are shared. Hence, deregulation of miRNA pathways extends well beyond the effects specific to *DGCR8* and applies broadly to patients with SAD or AOS carrying the 22q11.2 microdeletion and to patients with COS.

Given that each miRNA potentially downregulates up to hundreds of downstream targets [93], the researchers [76] further interrogated the mRNA expression profiles from iPSC-derived neurons (Table 3). Gene pathway and network analysis of differentially expressed genes (DEGs, *n* = 42) indicated a disruption of MAPK signaling in iPSC-derived neurons from patients with SCZ carrying the 22q11.2 microdeletion that may lead to perturbed neuronal proliferation and differentiation.

Beyond individual genes, weighted correlation network analysis (WGCNA) permits the detection of perturbed interactions between functionally interconnected genes that may represent only in part significant expression changes. In this study [76], WGCNA revealed, however, only subtle changes in 2 out of 15 gene modules identified. Accordingly, global wiring of functionally interconnected genes was unaffected in iPSC-derived neurons from patients. To uncover genes co-expressed with the DEGs, the researchers conducted a correlation analysis on different regions and developmental stages from human brain (i.e., BrainSpan database). Interestingly, DEGs were highly connected only during two developmental stages. The embryonic and the adolescent brain. Moreover, function enrichment analysis of the co-expression networks in the embryonic and adolescence brains showed that the embryonic cortex was enriched in genes critical to cell cycle, differentiation, and growth, while the adolescent cortex was enriched in genes critical to synaptic transmission and catabolism. In sum, these results support that a subset of the 22q11.2 microdeletion DEGs fulfill distinct functions during sensitive time-windows of brain development that become perturbed by haploinsufficiency.

The functional consequence of 22q11.2 haploinsufficiency for early neuronal and glial development has been assessed more recently by Toyoshima et al. [78] in neurosphere assays. These contain free-floating clusters of NSCs and provide a method to investigate iPSC-derived (Table 3) neural precursor cells in vitro. The researchers detected significant reductions in neurosphere size, neural differentiation efficiency, neurite outgrowth, and cellular migration in patient-derived cells. Although both patient- and control-derived neurospheres could be efficiently differentiated into neurons and astrocytes, the fraction of astrocytes among patient-derived differentiated cells was increased at the expense of neurons.

At the molecular scale, miRNA profiling [78] showed reduced expression of miRNAs belonging to the miR-17/92 cluster and miR-106a/b in patient-derived neurospheres. These miRNAs are predicted to target *MAPK14* transcripts encoding p38α, a member of the mitogen activated protein kinase family and regulator of neurogenic-to-gliogenic transition competence. Well-fitting this prediction, p38α was upregulated in patient-derived cells. Pharmacological inhibition of p38 in patient-derived neurospheres partially reinstated neurogenic competence. Moreover, mRNA expression profiling showed that DEGs between case/control neurospheres were enriched for genes relevant to cell differentiation, neuronal development, and microRNA processing. Specifically, upregulated genes in case neurospheres were significantly enriched for MAPK-mediated processes, neurotransmission, and signaling pathways. Collectively, these results indicate a ‘reduced neurogenic’ and ‘elevated gliogenic’ competence during early neurodevelopmental stages of patients with SCZ associated with a 22q11.2 microdeletion.

Taken together, different lines of evidence provide insight into the role of the 22q11.2 microdeletion as an early risk factor for AOS and COS: iPSC-derived neuronal cells show a delayed glutamatergic differentiation [73] and exhibit subtle deregulation of genes relevant for GABAergic, glutamatergic, and dopaminergic specification once they acquire electrical activities [74]. The effects of the 22q11.2 microdeletion appear to be mediated through different molecular mechanisms: an increase in the frequency of L1 insertion during early neurodevelopment through as yet unknown mechanisms may impair synaptic function and predispose for later disease [75]. Secondly, deregulation of miRNA pathways through *DGCR8* dependent and independent pathways control genes important to SCZ and neurodevelopment [76]. Such deregulation may disrupt MAPK signaling in iPSC-derived neurons from patients with SCZ and 22q11.2 microdeletion and lead to perturbed neuronal proliferation, differentiation, and increased gliogenic competence during early development [78]. Finally, deregulation of distinct coexpression gene networks at embryonic (cell cycle, differentiation, and growth) and adolescent (synaptic transmission and catabolism) stages.

### 5.2. Role of the 15q11.2 Microdeletion as Risk Factor for AOS and COS

The proximal long arm of chromosome 15 (15q11.2-q13) contains several CNVs that can increase the risk for common, severe neuropsychiatric disorders [101]. The CNVs arise from mis-paired low copy number repeats at three breakpoints denoted BP1, BP2, and BP3. The 15q11.2 BP1-BP2 microdeletion (Burnside-Butler syndrome) encloses four protein-encoding genes (*TUBGCP5*, *CFYIP1*, *NIPA1*, and *NIPA2*) and has a reported de novo frequency between 5–22%. On the other hand, about 35% and 51% of the carriers have inherited the microdeletion from an apparently affected or unaffected parent, respectively [102]. Genes inside the BP1-BP2 region are biallelically expressed, whereas the clinically related Prader-Willi/Angelman syndrome, defined by the distal breakpoint BP3 and the proximally located breakpoints BP1 or BP2, involves the deletion of a large genomically imprinted region between BP2-BP3.

Patients with the 15q11.2 BP1-BP2 microdeletion carry an increased risk for intellectual disability (ID), ASD, AOS, COS, and seizure disorders and manifest mild dysmorphic features and neurocognitive delay [102]. Discrete disabilities in learning, reading skills, and a marginally reduced intelligence quotient have been found among clinically affected, but also among normal individuals, with the 15q11.2 BP1-BP2 microdeletion [103]. Furthermore, this genetic variation affects brain structure in a pattern consistent with that observed during first-episode psychosis in SCZ [103].

The 15q11.2 BP1-BP2 microdeletion has a prevalence ranging from 0.57–1.27% as inferred from high resolution microarray analysis [102]. However, not all individuals with the deletion are clinically affected since this region harbors genetic material showing incomplete penetrance or low penetrance of pathogenicity along with variable expressivity. *NIPA1* (non-imprinted in Prader-Willi/Angelman syndrome 1 gene) is the best understood gene within this region and associates with autosomal dominant hereditary spastic paraplegia. It is highly expressed in neuronal tissues and serves the transport of Mg^2+^. Likewise, NIPA2 (non-imprinted in Prader–Willi/Angelman syndrome 2 gene) regulates renal Mg^2+^ transport. The *TUBGCP5* (tubulin gamma complex associated protein 5) gene is required for microtubule nucleation at the centrosome and is thought to contribute to neurobehavioral disorders such as ADHD (attention deficit hyperactivity disorder) [104]. Finally, cytoplasmatic FMR1-interacting protein (CYFIP1), a binding partner of fragile X mental retardation protein (FMRP), is a leading candidate inside the BP1-BP2 domain. CYFIP1 has been found to interact with Rac1 (a RHO GTPase involved in modulation of the cytoskeleton, neuronal polarization, axonal growth, and differentiation), FMRP, and EIF4E (eukaryotic translation initiation factor 4E). In mice, complex formation between cyfip1, FMRP, and cap protein eiF4E serves to regulate activity-dependent protein translation in mature neurons [105]. Biochemical studies further suggest that CYFIP1 regulates the WAVE complex that controls Arp2/3-medited actin polymerization and membrane protrusion formation in non-neuronal cells.

15q11.2 microdeletion is one of the most frequent CNVs associated with an increased risk for AOS and COS (Table 1) [49]. To understand why 15q11.2 CNVs are prominent risk factors for SCZ, Yoon et al. [79] established iPSC lines from three individuals with COS carrying the microdeletion, and from five healthy individuals without the microdeletion (Table 3).

Immunostaining of iPSC-derived neural rosettes (an in vitro pendant of the neural tube) from COS cases displayed perturbed apical–basal polarity and disrupted adherens junctions relative to controls. The actin cytoskeleton acts as a cytoplasmatic anchor for cadherin/catenin proteins at adherens junctions and its proper organization is important for maintaining adherens junctions and polarity of NPCs. Consistent with CYFIP1’s role as a regulator of the actin-modulating WAVE complex, biochemical analysis showed a specific defect of WAVE complex stabilization in NPCs carrying the 15q11.2 microdeletion. Gain-and-loss of function experiments for CYFIP1 in NPCs carrying the microdeletion and from control NPCs further supported this finding. In agreement with the in vitro experiments, cyfip1 was also necessary to sustain adherens junctions and apical polarity of NSCs in the developing mouse cortex as demonstrated by in vivo knockdown experiments. Moreover, deficits in cyfip1 led to false placement and pattern of mitosis of radial glial progenitor cells (RGCs) in the developing mouse cortex. This phenotype subsisted in intermediate progenitor cells (IPCs), the direct progeny of RGCs, as well as in glutamatergic projection neurons, resulting in cortical layer malformation.

Beyond NSC/NPC, the function of CYFP1 is known to extend to mature neurons. Recent reports showed that cyfip1 is enriched at mouse neuronal synapses and plays an important role in dendritic arborization as evidenced by gain-and-loss of function studies [106,107]. Given that human postmortem studies support a role for dendritic spine structure abnormalities in the pathogenesis of ID, ASD, and SCZ [108], haploinsufficiency of *CYFIP1* could present a mechanism whereby the 15q11.2 deletion confers risk for neuropsychiatric disorders. To address this topic, Das et al. [80] created iPSCs from a mother and her offspring, both carrying the 15q11.2 deletion, and a control with an intact chromosome 15. The offspring, but not the mother, additionally manifested SAD.

Neural rosettes derived from quality controlled iPSCs (Table 2) were dissected, expanded as neurospheres, subsequently kept as monolayers, and finally differentiated into neurons (Table 4). The expression of all four genes inside the deleted region as well as of PSD95, a key marker of synapses, was reduced during different stages of neuronal development in the mother and offspring when compared to the unrelated control. Moreover, at 10 weeks of differentiation qualitative analysis of iPSC-derived neurons provided tentative evidence that dendritic morphology was altered in 15q11.2-deletion carriers relative to control. In support of this view, Dimitrion et al. [109] observed in a follow up study that in low-density neuronal cultures the density of dendritic filopodia was strongly increased in neurons with the microdeletion (i.e., the maternally-derived iPSC line) relative to the control.

Collectively, these studies show that human iPSCs can serve as an entry point to investigate a common CNV risk factor for AOS, COS, and other neuropsychiatric disorders. Results from multiple levels of analysis also allowed prioritization of genes within the CNV and highlighted a role of CYFIP1 as contributing factor to biological processes implicated in the neurodevelopmental origins of these disorders. Specifically, CYFIP1 regulates apical–basal polarity and adherens junctions of NSCs, proper positioning of NSCs and their derivatives along neurodevelopmental trajectories, and dendritic arborization of mature neurons; all of these processes are key to AOS and COS.

### 5.3. Role of the 2p16.3 Microdeletion as Risk Factor for AOS and COS

AOS and COS has been associated with non-recurrent CNVs (Table 1) including those disrupting the *NRXN1* gene at 2p16.3. These deletions cluster in delineated regions and represent with variable size and unique breakpoints. The presence of short stretches of microhomology and additional base pair insertions at the breakpoint site [110] suggests that error-prone repair mechanisms referred to as NHEJ bridge, modify, and fuse free DNA ends at sites of double-stranded chromosomal breaks. In contradiction to NAHR, NHEJ does not depend on specific genomic architectural features such as LCR.

*NRXN1* encodes neurexin-1 [111], an evolutionary conserved presynaptic cell-adhesion molecule. Humans contain three neurexin genes (*NRXN1*, *NRXN2*, and *NRXN3*) each of which harbors separate promoters for longer α- and shorter β-neurexins. These isoforms bind to postsynaptic cell-adhesion molecules such as neuroligins and LRRTMs that are also associated with ASD or SCZ.

Most *NRXN1* mutations represent heterozygous CNVs that delete only *NRXN1* due to the large size of the gene, while missense and truncation mutations are less frequent [110]. While *NRXN1* mutations are rare (≈0.18% of patients with SCZ [112]), they represent the most frequent-single gene mutation in AOS and COS. *NRXN1* polymorphisms have been also implicated in differential responses to antipsychotic medication in SCZ further strengthening the link between SCZ and *NRXN1* [113]. Individuals with 2p16.3 microdeletion can manifest developmental delay, especially in speech, abnormal behaviors, and mild dysmorphic features with epilepsy [114]. However, presence of *NRXN1* deletions in healthy parents and siblings indicates reduced penetrance and/or variable expressivity.

The variable clinical presentations and the observation that homozygous *Nrxn1α* mutations cause only a minor phenotype in mice [115], raise the question of whether heterozygous *NRXN1* mutations alone directly impair synaptic function. To address this question under conditions that control precisely for genetic background, Pak et al. [81] established isogenic human embryonic stem cell (ESC) lines carrying different heterozygous conditional *NRXN1* mutations and analyzed subsequently their effects on neuronal phenotypes and activities.

Loss-of-function mutations were generated by homologous recombination and consisted either of a conditional exon deletion that caused a frameshift and disrupted both neurexin-1α and -1β or a conditional truncation of neurexin-1α and -1β that introduced a stop codon and resulted in rapidly degraded protein. Both heterozygous conditional *NRXN1* mutations did not alter the electrical properties of human neurons, their synapse numbers, or dendritic arborization. Yet, they produced a severe and selective decrease in presynaptic neurotransmitter release concomitant with a reduction in spontaneous mEPSC (miniature excitatory postsynaptic current) frequency, but not amplitude, and a parallel decrease in evoked EPSC amplitude. Interestingly, the decrease in EPSC amplitude was rapidly relieved during a stimulus train indicating that this phenotype did not involve a general decline of the release probability, but exhibited a specific decrease in release probability for only the first stimulus. Moreover, key features of the *NRXN1* heterozygous mutant phenotype were detected in two different types of ESC-derived human cells: induced neurons (iN) consisting of a homogenous population of excitatory forebrain neurons, and a more heterogeneous population of neurons obtained from an NPC intermediate (Table 4). This observation strengthens the notion that heterozygous loss of *NRXN1* causes a selective impairment in synaptic transmission. A plausible explanation for this phenotype is impairment in presynaptic Ca^2+^ influx during an action potential that would only initially impair release in a high-frequency stimulus train due to the accumulation of residual Ca^2+^ later in the train.

Collectively, these results suggest that heterozygous *NRXN1* mutations may predispose to AOS, COS, and other neuropsychiatric disorders by impairing a highly specific synapse function.

### 5.4. Role of the 16p11.2 Microdeletion as Risk Factor for AOS and COS

The 16p11.2 CNV covers an ≈600 kb locus encompassing 29 annotated genes [116]. Carriers with either the deletion (16p-del) or the duplication (16p-dup) of this region manifest psychiatric disorders such as ASD, AOS, and COS (Table 1). Common developmental, cognitive, and behavioral symptoms are also equally shared by both genotypes. By contrast, they associate with opposing physical symptoms: individuals with 16p-del have normal birth weight, but develop a drastic increase in body mass index (BMI) by age 7 such that ≈75% of adult carriers are obese. Contrariwise, individuals with 16p-dup represent with below-normal weight at birth and an eightfold enhanced risk of underweight in adulthood. Additionally, carriers differ in head sizes: ≈17% of the individuals with 16p-del are macrocephalic, while ≈10% of the individuals with 16p-dup are microcephalic [116]. Neuroimaging studies on carriers suggest significant effects on gray matter volume, especially increase in the cortical surface area in individuals with 16p-del. On the other hand, a reciprocal decrease has been detected in individuals with 16p-dup [117]. Noteworthy, Lin et al. [118] predicted by dynamic protein interaction analysis profound changes in the 16p11.2 protein interaction networks throughout different stages of brain development and/or in different brain regions. Hereby, the late mid-fetal period of cortical development was most critical for establishing the connectivity of 16p11.2 proteins with their co-expressed partners.

To uncover cellular phenotypes due to 16p11.2 CNVs, Desphande et al. [82] generated iPSCs from donors with a diagnosis of ASD with gain (dup) or loss (del) of 16p11.2 CNV (Table 3). Quality control (Table 2) confirmed that 16p11.2 CNV carrier-derived iPSCs were comparable to control iPSCs regarding pluripotency, NPC proliferation, self-renewal, and the formation of forebrain neurons. By contrast, at three and six weeks post differentiation, 16p del-derived neurons showed neuronal hypertrophy with increases in soma size, total dendrite length and arborization, whereas 16p dup-derived neurons showed the opposite phenotype relative to controls, especially in excitatory neurons.

Functionally, 16p del-derived neurons exhibited reduced excitability with greatly reduced voltage responses and membrane resistance relative to 16p dup-derived neurons, which behaved undistinguishably to controls. On the other hand, 16p dup-derived neurons—but neither 16p del-derived neurons nor controls—showed an increased potassium current density at positive voltages indicating that they may compensate for their reduced somatic size by increasing the outward potassium current to stabilize intrinsic excitability. Finally, both 16p del- and dup-derived neurons revealed a lower density of excitatory synapses compared with controls that associated with a significant increase in the amplitude, but unaltered kinetic or frequency, of mEPSCs.

Collectively, reciprocal cellular phenotypes in 16p-dup/del iPSC-derived neurons may contribute to opposing brain size difference. In this respect a gene inside 16p11.2, namely *KCTD13*, encoding a nuclear protein that stimulates DNA polymerase activity at replication foci, has been shown to cause via proliferation dose-dependent macrocephaly in zebrafish [119]. Furthermore, KCTD13 plays a crucial role in the regulation of the KCTD13-Cul3-RhoA pathway in layer 4 of the inner cortical plate that controls brain size and connectivity [118]. At the same time, similar reductions in synapse density in either 16p11.2 genotype may contribute to the similarities in human clinical outcome and represent a major risk factor for the development of SCZ—the ‘disease of the synapse’ [120].

### 5.5. COS with or without CNVs

Despite the rare incidence of COS, recent studies [83,84,85] have taken a step forward toward the collection of larger sample sizes from patients with COS carrying risk CNVs and from those without known CNVs (i.e., idiopathic COS). In 2016, Topol et al. [83] reported the first COS/control study comprising each ten individuals: Patients with COS carried different CNVs (1p33, 2p16.3 del, 3p25.3, 16p11.2, and 22q11.2) or showed no detectable anomalies (*n* = 4) (Table 3). These patients were recruited from the longitudinal NIHM study (see Section 2) and showed across development reduced cortical thickness relative to controls. With increasing age developmental trajectory normalized in parietal regions but remained divergent in frontal and temporal regions, a pattern of loss similar to AOS [27].

Quality controlled COS/control iPSCs (Table 2) were differentiated via dual-SMAD inhibition into NPCs (Table 4), expanded, and harvested for miRNA profiling (Table 5). As noted before (Section 5.1), miRNAs play a pivotal role in the developing human brain [91,92] and altered miRNA expression profiles have been consistently detected in psychiatric disorders [94]. In parallel, the researchers conducted miRNA expression profiling also on previous AOS/control samples [61]. Among 800 miRNAs detected by digital expression profiling (Nanostring), miR-9, a regulator of neurogenesis in NSCs [121], was the most abundant and the most downregulated miRNA in NPCs from patients with either AOS or COS. Thereby, lower miR-9 levels in patient-derived NPCs relative to those from controls were largely driven by a subset of cases, which is not unexpected given the heterogeneity of a complex disorder like SCZ. Functionally, miR-9 enhanced radial migration as evidenced by gain-and-loss of function experiments in iPSC-derived NPCs.

Analysis of the AOS/control cohort, for which mRNA expression profiles from both iPSC-derived NPCs and neuronal cells were already available [61], suggested that known miR-9 target genes were significantly enriched (*n* = 84) among DEGs (56% upregulated, 44% downregulated). In this context it is interesting to note that previous SCZ GWAS gene-set enrichment analysis [43] has detected an enrichment on predicted miR-9 targets among SCZ-associated genes [122]. Together, these findings indicate that genetic variants in both miR-9 and its targets confer increased risk of SCZ.

Moving beyond miRNA profiling, the same case/control cohorts were also investigated for the expression of the brain-specific tyrosine phosphatase STEP (striatal-enriched protein tyrosine phosphatase) [84]. This membrane associated kinase is an important regulator of synaptic function: it counteracts synaptic strengthening by enhancing *N*-methyl-d-aspartate glutamate receptor (NMDAR) internalization through phosphorylation of the GluN2B subunit and inactivation of the extracellular signal-regulated kinase 1/2 and Fyn. Previous studies suggested that STEP_61_ is higher expressed in postmortem anterior cingulate cortex and dorsolateral prefrontal cortex of patients with AOS, as well as in mice treated with the psychomimetic phencyclidine or the NMDAR antagonist MK-801 [123].

In a separate approach, the researchers [84] had originally found enhanced expression of STEP_61_ in the cortices of Nrg1^+/−^ (Neuregulin 1) and brain-specific ErbB2/4 knockout mice. Nrg1 signaling is a critical mediator of synaptic function and plasticity in glutamatergic signaling [124]. Therefore, Nrg1^+/−^ knockout mice are deemed a valued translation model of SCZ.

In support of these findings, STEP_61_ protein expression was also increased relative to controls in mixed or merely pure glutamatergic forebrain cultures generated from AOS- or COS-derived iPSCs. Similar to miR-9 expression, differences in STEP_61_ protein expression were driven by a subset of patients with AOS (three out of four) or patients with COS (four out of nine) collaborating previous evidence for genetic heterogeneity in either cohort [83]. Notably, knock-down or pharmacological inhibition of STEP prevented the loss of NMDARs in iPSC-derived neurons from patients with AOS or mice brain and normalized behavior in Nrg1^+/−^ mice.

Collectively, findings from transgenic mice models and patient-specific iPSCs support perturbed glutamate signaling in AOS and COS, and thus attest to the glutamate hypothesis of SCZ [125].

In a most recent study [85], an enlarged collection of COS/control iPSCs (Table 2 and Table 3) was differentiated into NPCs and forebrain neurons to carry out mRNA sequencing (RNA-seq). A rigorous bioinformatic strategy was applied to adjust for technical variation and batch effects, spurious samples and samples that showed aberrant X-inactivation or contamination. Despite these precautions, the researcher observed large heterogeneity in cell type composition (CTC) between NPCs and neurons, even from the same individual. This indicates that differences in differentiation capacity led to unique neural compositions in each sample. Computational deconvolution analysis of CTC helped sharpening the distinction between NPCs and neurons; however, substantial heterogeneity remained, partly due to neural crest and mesenchymal contaminants. In fact, variation due to cell type heterogeneity surpassed variation due to donor effects and represented an important source of intra-donor expression variation that could hamper the analysis of inter-donor variation (i.e., case/control differences).

Owing these limitations, differential expression analysis of NPCs and neurons generated from COS- and control-derived iPSCs identified only few genes: one gene (*ENSG00000230847*; Occludin pseudogene) with FDR < 10% and one gene (*FZD6*, Frizzled Class Receptor 6) with FDR < 30% were both shared by NPCs and neurons. An additional three genes (*GTF2H2B*, General Transcription Factor IIH Subunit 2; *ELTD1*, EGF, latrophilin and seven transmembrane domain containing 1; *ENSG00000236725*, pseudogene RP11-154P18.1) with a FDR < 30% were specific to NPCs. Conversely, another three genes (*QPCT*, Glutaminyl-peptide cyclotransferase; *CBX2*, Chromobox homolog 2, drosophila Polycomb class; *INTS4P1*, integrator complex subunit 4 pseudogene 1) with a FDR < 30% were specific to neurons. Although plausible candidates in COS pathology such as FZD6, QPCT, and CBX2 were differentially expressed, no coherent set of biological pathways could be identified.

Moving beyond iPSCs, Hoffman et al. [85] therefore analyzed the concordance between gene expression in iPSCs-derived cells from patients with COS and differential expression results from post-mortem brain case/control studies from five psychiatric diseases: Alcoholism, MDD, BP, SCZ, and ASD. High concordance was observed for SCZ (higher in neurons vs. NPCs), BP, and ASD. By contrast, concordance was low for alcoholism and MDD. This outcome supports the specificity of gene expression data from iPSC-derived cells from patients with COS and agrees with current insight on cross-disorder genetic liability of psychiatric disorders [95,126].

Collectively, iPSC-based case/control studies on patients with COS have provided further insight into potential neurodevelopmental deviations. Reduced miR-9 expression in a subset of samples points to impaired radial migration of NPCs during early steps of development. In support of this view, increased STEP_61_ protein expression in NPCs from patients with COS suggests perturbed glutamate signaling. Neuronal migration in the cortex is controlled by the paracrine action of the classical neurotransmitters glutamate and GABA (γ-aminobutyric acid) [127]. Glutamate controls radial migration of pyramidal neurons by acting primarily on NMDA receptors and regulates tangential migration of inhibitory interneurons by activating non-NMDA and NMDA receptors. In general, intra-donor and inter-donor differences in differentiation capacity of iPSCs can obscure detection of disease-relevant signals in case/control studies. However, subtle though statistically significant concordance between both NPCs and neurons generated from iPSCs derived from patients with COS and two recent SCZ post-mortem cohorts supports that in vitro findings can recapitulate processes from the diseased brain, at least in part.

## 6. Future Perspectives and Challenges

The possibility to generate patient-specific iPSCs has provided unique opportunities for the investigation of living disease-relevant cells from patients with COS and associated genetic risk factors. iPSC-based studies on CNVs associated with AOS and COS has helped to advance our insight in the biological underpinnings of these variations: CNVs enhance L1 retrotransposition to synaptic genes during early neurodevelopment [75] and perturb miRNA expression [76,78] thus contributing to impaired mitogenic signaling [77,78]. Furthermore, CNVs disrupt the formation of adherens junctions and apical polarity in early NPCs, especially RGCs, with long term effects on cortical organization [79]. The effects of CNVs on NPCs and neuronal cells are manifold; they reach from more subtle alterations in the expression of synaptic markers and dendritic morphology [80] to overt differences in NPC soma size, arborization, and excitatory synapses [82]. These morphological changes concur with distinct and selected changes in electrical activity such as reduced frequency [81] or increased amplitude of mEPCs [82]. In addition, more recent case/control studies on patients with COS have highlighted perturbed miRNA expression potentially affecting neurogenesis, radial glia migration [83], and glutamate signaling [84]. Importantly, transcriptional signatures of iPSC-derived NPCs and neurons from patients with COS show concordance with postmortem case/control samples from SCZ, but also with genetically related BP and ASD, and indicate that changes observed in vitro may reflect changes from the diseased brain. Unsurprisingly, there is no one-fits-all cellular or molecular phenotype emerging from these studies. While this is likely owed genetic heterogeneity in COS, it also raises questions as to the different differentiation protocols applied in current iPSC studies, especially, as to cellular heterogeneity that may obscure detection and reproducibility of disease-specific signals within and across COS/control studies. Therefore, we discuss next current caveats and further steps to be taken to improve the generation and design of patient-specific iPSC studies for COS and beyond.

### 6.1. High Resolution Karyotypes

Random mutations can arise along the reprogramming process and/or during in vitro culture at any time. Nowadays, non-integrating, so-called ‘foot-print free’, reprogramming techniques (i.e., Sendai virus, episomal, and mRNA transfection) (Table 2) are the method of choice to guard against random integration into the host genome. However, these techniques are not perfect: SNP array systems with an average genomic resolution of 43 KB (as opposed to 5 MB by traditional G-banding) showed the highest aneuploidy for retroviral (13.5%) and episomal (11.5%) derived iPSCs [128]. In-between aneuploidy was detected for lentiviral (4.5%) and Sendai virus (4.6%) derived iPSCs, and lowest aneuploidy for RNA (2.3%) derived iPSCs. Furthermore, whole exome sequencing suggests that clonal fibroblasts and iPSCs derived from the same fibroblast carry a similar number of mutations [129]. Accordingly, more than 90% of the mutations preexist randomly in small subsets of the parental unselected fibroblast population. Common genetic variations underpin molecular heterogeneity in iPSCs [130,131,132,133,134,135] and any genetic variation arising during reprogramming or in vitro culture can have potentially the same effect. Only recently, studies on COS (Table 2) have sought for donor-matched digital (e.g., SNP-based) karyotype maps to assess chromosomal anomalies, including copy number alterations [133], more precisely.

Digital karyotyping, but also mRNA-sequencing [85], can inform additionally on familial relationships and the proper assignment of iPSC lines and should be implemented in future iPSC studies on a routine basis.

### 6.2. Cellular Heterogeneity

Randomly distributed differences in genotype, expression profiles, and epigenetic state of individual iPSC lines [136] are known to influence the (neural) differentiation capacity of human embryonic stem cells and iPSCs from healthy donors [137,138,139,140]. Predictably, such variations will confound our ability to identify those related to disease status in a case/control design. Recent iPSC studies have therefore aimed to clarify to what degree variance across donors explains expression variation: Carcamo-Orrive [132] observed that ≈50% of genome wide expression variability in undifferentiated iPSCs (317 iPSCs from 101 healthy individuals) is explained by genetic variation across individuals. They also identified Polycomb targets to contribute significantly to the non-genetic variability seen within and across individuals [141]. By means of genome-wide profiling, Kilpinen et al. [133] determined that 5–46% of the variation (variation median ≈6) in different iPSC phenotypes (711 iPSCs from 301 healthy individuals), including differentiation capacity and cellular morphology, arise from differences between individuals. Relatedly, Schwartzentruber et al. [142] observed that sample-to-sample (*n* = 123) variability in gene expression in iPSC derived sensory neurons from healthy donors clearly surpassed the one from in vivo dorsal root ganglia. Thereby, levels of variation for donor and reprogramming (23.2% in aggregate) were close to those from neuron differentiation batch (24.7%) reflecting varying mixtures of cell types across differentiation. Lastly, in a genetically heterogeneous and small cohort of patients with COS, Hoffman et al. [85] measured a smaller donor effect (2.2%) in iPSC-derived neurons that were obtained either by directed differentiation (dual-SMAD inhibition), known to give rise to various neuronal cell types, or by induced differentiation (Ngn2 overexpression), leading to mostly excitatory forebrain neurons (Table 4).

Recent advancements can help improving analysis of cellular heterogeneity in future case/control studies: induced neurons (iN) [143,144,145,146], generated by lentivirus-mediated overexpression of selected neuronal transcription factors, offer the benefit of less heterogeneous cell populations that may allow to detect more subtle albeit highly significant effects (e.g., [81]). Although iNs are more homogeneous with respect to cell type, they continue to display variable maturity as detected by single cell sequencing [143]. As an alternative to FACS-sorting, reporter gene assays can serve to select highly differentiated neurons with increased functionality for electrophysiology or transcriptional profiling [143]. Right now, large scale analysis of case/control samples by single cell sequencing appears still cost-prohibitive to most laboratories. In this situation, dissecting transcriptomic signatures of neuronal differentiation and maturation by improved computational skills (i.e., cellular deconvolution) may offer a more feasible alternative [62].

Implementation of these measures can help to substantially reduce or resolve cellular heterogeneity for improved detection of disease-specific signals. However, such improvements do not necessarily help to distinguish truly disease associated changes in (endo-) phenotypes from random line and culture artifacts. Testing of multiple cell clones per donor and of different differentiation protocols for the generation of the same or different cell types is strongly recommended, once preliminary results are obtained in case/control studies. Along the same line, postmortem analysis of case/control brain samples, despite known inherent limitations, is an important approach to collaborate iPSC-based findings [85]. Ideally, postmortem brain samples are not processed as bulk tissue, but as single cells, particularly for transcriptomics, to avoid anew pitfalls from cellular heterogeneity [147]. While still a matter of ongoing debate, these strategic guidelines can enhance the quality of iPSC studies on COS we should look for in the future.

### 6.3. Polygenic Disorders and the Environment

The presence of rare, highly penetrant genetic variants that associate with distinct cellular and molecular defects is a hallmark of Mendelian disorders. On the other hand, the basis of polygenic disorders such as COS is still less understood with numerous (non-) coding variants of small effect size converging jointly with rare variants of large effect size on highly complex phenotypes of varying expressivity. Despite this challenge, present iPSC studies on CNVs associated with AOS and COS have provided valuable information on cellular and molecular phenotypes (Table 5) of potential relevance to early neurodevelopment. Yet, given the small number of donors, for both cases and controls [148], we have to ask to what degree these observations can be generalized or specify only a subset of patients. In fact, iPSC-studies on patients with COS suggested considerable heterogeneity between phenotypes in vitro such miRNA expression [83] and glutamate signaling [84].

Although genetically-informed selection for patients with SCZ is thought to benefit detection of disease relevant signals in heterogeneous cell samples, or even to reduce cellular heterogeneity during differentiation, the size of cohorts needed to reach this goal is still a matter of uncertainty [148]. Schwartzentruber et al. [142] have provided provisional insight on this issue: they identified thousands of quantitative trait loci regulating gene expression, chromatin accessibility, and RNA splicing during neuronal differentiation in a large iPSC-derived cell sample (*n* = 123). In light of this finding, iPSCs from 20–80 donors appear sufficient to detect the effect of common regulatory variants of moderate to large effect sizes. Remember, that effect sizes of certain CNVs associated with SCZ are among the strongest known so far for this disorder suggesting that cohort sizes needed for genetically-stratified patients with COS are probably in the lower range of this estimate.

SCZ is a highly heritable (see Section 3); however, environmental risk factors are likewise important to SCZ pathogenesis. Insight into the mechanisms mediating the interaction of risk genes with environmental risk factors remains an important endeavor to attain a more comprehensive picture of this disease. Since the effects of environmental factors on specific disease-relevant cell-types cannot assessed in living patients, iPSC-based studies may provide a tractable model for this purpose. According to the neurodevelopmental hypothesis of SCZ, early deviations may stay latent until called into operation through maturational processes. In analogy, many SCZ-associated processes may be hidden in simple monolayer iPSC-derived NPC/neuron cultures and may be only detected through activity-dependent processes arising from neuronal-activity or transcriptional activation in response to stimuli mimicking environmental exposures. For example, exposure of iPSC-derived neurons to Δ9 tetrahydrocannabinol (THC, a major compound of cannabis), either acutely or chronically, dampened the neuronal transcription response following depolarization and was associated with significant synaptic, mitochondrial, and glutamate signaling alterations [149]. While the final verdict about the causal nature of the cannabis–psychosis association is still out [150], we may attain nevertheless a better understanding of its potential cellular and molecular underpinnings from iPSC-based studies.

### 6.4. Organoids—From Structure to Function?

As of yet, current differentiation protocols applied to iPSC-based case/control studies on COS do not recreate the three-dimensional organization of the human brain and well-known structure-function relationships [151]. Remember, MRI studies on patients with COS implicated greater global connectedness concomitant to impaired short-range connectivity and disrupted modularity [29,30]; a pattern barely portrayed in 2D-culture.

Recent advances on the generation of region-specific brain organoids is anticipated to address this challenge, at least in part [152,153]. In this approach, pluripotent cells are used to reproduce in vitro key aspects of human brain development and function within three-dimensional structures termed ‘brain organoids’. As the name suggests, ‘brain organoid’ is not the same as a ‘brain’, but represents a reductionist cellular system that recapitulates some aspects of the cellular composition and activity of the brain, and that in its generation follows at least some of the steps of early human embryonic brain development. Although today’s brain organoids can give rise to active neurons and functional circuits, they do not match the anatomical organization or connectivity of the living brain [152,153]. At the same time, organoid-to-organoid variability in architecture and cell-type composition imposes as yet a severe hurdle on case/control studies. In a nutshell, brain organoids are presently barley suited as first-line screening tool in iPSC-based case/control studies, but may allow deepening insight into findings from well-defined monolayer cultures in a model closer to neurodevelopment in vivo. As an alternative approach to organoids, transplantation of iPSC-derived neuronal cells into embryonic or adult mice may help to recapitulate the physiology of SCZ more closely than 2D culture, and ideally highlight associated behavioral phenotypes. In support of this view, iPSC-derived cortical neurons from patients with Down syndrome showed increased synaptic stability and reduced oscillation relative to controls when transplanted in the adult mouse cortex [154].

All in all, COS remains a major challenge with earlier onset, more severe course, and poorer outcome relative to AOS. The need for an improved understanding of the cellular and molecular underpinnings of COS pathology persists despite recent progress on genetics, neuroimaging, and therapy. The transformative discovery of iPSCs [7] has paved the way for new translational strategies to trace early neurodevelopment deviations of COS in vitro. iPSC-based studies on patients with COS do not recreate the complex cellular and spatio-temporal phenotypes from the perinatal and adult brain, nor do they mimic early or late clinical symptoms of patients with COS in a dish. However, they create new opportunities to deliver actionable knowledge, i.e., genetic findings whose biological implications can be used to improve diagnosis, to develop rationale therapies, and craft mechanistic approaches to primary prevention. For example, iPSC-based disease modeling has led to drug repurposing [155] in amyotrophic lateral sclerosis (ALS): Hyperexcitability of iPSC-derived motor neurons from patients with ALS could be reversed by retigabine resulting in better survival of ALS motor neurons. Previously approved by the Federal Drug Administration FDA for the treatment of epilepsy, retigabine is now in clinical trial in ALS, encouraging the effort to use iPSC-derived models for development of new therapies, including drug screening, drug repurposing, and tailored treatments [156] for patients with COS. Beyond present progress, generation of iPSC-derived living neurons from patients with COS will not only transform our mindscape of this disease, but can also help to improve the lives of patients and their families.

## Figures and Tables

**Table 1:** Significant CNV loci in patients with AOS and COS.

Chr	Locus	Mechanism	CNV	Effect	OR (95% CI)	COS
1	1q21.1	NAHR	Loss + gain	Risk	3.8 (2.1–6.9)	
2	2p16.3 (*NRXN1*)	NHEJ	Loss	Risk	14.4 (4.2–46.9)	+
3	3q29	NAHR	Loss	Risk	Infinite	+
7	7p36.3	NAHR	Loss + gain	Risk	3.5 (1.3–9.0)	
7	7q11.21	NAHR	Loss + gain	Protective	0.66 (0.52–0.84)	
7	7q11.23	NAHR	Gain	Risk	16.1 (3.1–125.7)	
8	8q22.2	NHEJ	Loss	Risk	14.5 (1.7–122.1)	
9	9p24.3	NHEJ	Loss + gain	Risk	12.4 (1.6–98.1)	
13	13q12.11	NAHR	Gain	Protective	0.36 (0.19–0.67)	
15	15q11.2	NAHR	Loss	Risk	1.8 (1.2–2.6)	+
15	15q13.3	NAHR	Loss	Risk	15.6 (3.7–66.5)	+
16	16p11.2. proximal	NAHR	Gain	Risk	9.4 (4.2–20.9)	
16	16p11.2. distal	NAHR	Loss	Risk	20.6 (2.6–162.2)	+
22	22q11.21	NAHR	Loss	Risk	67.7 (9.3–492.8)	+
22	22q11.21	NAHR	Gain	Protective	0.15 (0.04–0.52)	
X	Xq28	NAHR	Gain	Protective	0.35 (0.18–0.68)	
X	Xq28. distal	NAHR	Gain	Risk	8.9 (2.0–39.9)	

Abbreviations are: AOS, adult-onset schizophrenia; Chr, chromosome; CI, confidence interval; COS, childhood-onset schizophrenia; CNV, copy number variation; NAHR, non-allelic homologous recombination; NHEJ, non-homologous end joining; OR, odds ratio; +, present. Adapted by Springer Nature (https://www.nature.com/nature/), Contribution of copy number variants to schizophrenia from a genome-wide study of 41,321 subjects, Christian R. Marshall, 2017 [49].

**Table 2:** iPSC generation and quality control.

Ref	Source	Factors	Method	*n*	Authentication	Karyotype	Pluripotency
[73]	Fibroblast	OKSM	RV	-	-	G-B, F	ICC, EB
[74]	As in [73]	OKSM	RV	-	-	G-B, F	ICC, EB
[75]	Fibroblast	OKSM	RV	-	-	CGH	ICC, Tera, EB
[76]	Fibroblast	OKSML	Epi	add	-	G-B, F	ICC, EB
[77]	As in [76]	OKSML	Epi	add	-	G-B, F, micro	ICC, EB
[78]	As in [75]	OKSM	RV	add	-	CGH, Taq	ICC, Tera, EB
[79]	Fibroblast	OKSML	Epi or Sen		-	G-B, F	ICC, Tera
[80]	Fibroblast	OKSM	Sen	add	-	G-B, CGH	ICC
[81]	hESC (H1)	na	na	-	-	na	na
[82]	Fibroblast	OKSML	Epi	na	CytoChip SNP	CGH, SNP	ICC
[83]	Fibroblast	OKSM	Sen	2–3	PsychChip SNP	G-B	FACS, PCR
[84]	As in [83]	OKSM	Sen	2–3	PsychChip SNP	G-B	FACS, PCR
[85]	As in [83]	OKSM	Sen	2–3	Verif-BamID [86]	G-B	FACS, PCR

Abbreviations are: add, additional iPSC clones for some donors; EB, embryoid body formation combined with ICC and/or qPCR; Epi, episomal plasmid; CGH, comparative genomic hybridization microarray; G-B, chromosomal G-banding; FACS, fluorescence activated cell sorting; F, fluorescence in-situ hybridization; ICC, immunocytochemistry; micro, microarray; *n*, numbers of independent clones per donor; na, non-applicable; OKSM, reprogramming factors OCT4, KLF4, SOX2, MYC; OKSML, reprogramming factors plus Lin28 and p53 shRNA; PCR, quantitative reversed transcribed polymerase chain reaction; Sen, Sendai virus; Ref, reference; RV, retroviral transduction; SNP, single nucleotide polymorphism; Taq, Taqman copy number assay; Tera, teratoma formation.

**Table 3:** Study design, cellular model, and neuronal cell types.

Ref	Case/Control	Deletion	Model	Major Cell Type
[73]	AOS (*n* = 1)	22q11.2	iPSC	Forebrain glutamatergic neurons
	AOS (*n* =1) COS (*n* = 1) Ctr (*n* = 2)	- - -		
[74]	AOS (*n* = 1) [73]	22q11.2	iPSC	Early post-mitotic neurons
	Ctr (*n* = 1) [73]	-		
[75]	AOS (*n* = 2)	22q11.2	iPSC	Mixed early neuronal and glial cell types
	Ctr (*n* = 2)	-		
[76]	AOS (*n* = 1)	22q11.2	iPSC	Mixed early glutamatergic and GABAergic neurons
	SAD (*n* = 3)	22q11.2		
	COS (*n* = 2)	-		
	Ctr (*n* = 6)	-		
[77]	As in [76]	As in [76]	iPSC	As in [76]
	+ COS (*n* = 2)	-		
	+ Ctr (*n* = 1)	-		
[78]	As in [75]	As in [75]	iPSC	As in [75]
	+ Ctr (*n* = 1)	-		
[79]	COS (*n* = 3)	15q11.2	iPSC	Rosette-derived cortical NPCs
	Ctr (*n* = 5)	-		
[80]	SAD (*n* = 1)	15q11.2	iPSC	Rosette derived neurons
	Mother (*n* = 1)	15q11.2		
	Ctr (*n* = 1)	--		
[81]	Isogenic hESCs	Mutated heterogeneous	hESC	Induced glutamatergic neurons, mixed forebrain neurons
		*NRXN1* alleles		
[82]	ASD (*n* = 1)	16p11.2 dup, de novo	iPSC	NPCs, dorsal forebrain neurons, up to 14 weeks maturated
	NSD (*n* = 1)	16p11.2 dup, de novo		
	NSD (*n* = 1)	16p11.2 dup, inherited		
	Autism (*n* = 1)	16p11.2 del, de novo		
	Autism (*n* = 1)	16p11.2 del, unknown		
	Autism (*n* = 1)	16p11.2 del, inherited		
	Ctr (*n* = 4)	-		
[83]	COS-1 (*n* = 1)	1p33	iPSC	NPCs
	COS-2 (*n* = 1)	2p16.3 del (*NRXN1*)		
	COS-3 (*n* = 1)	3p25.3		
	COS-4 (*n* = 2)	16p11.2		
	COS-5 (*n* = 1)	22q11.2		
	COS-6 (*n* = 4)	-		
	Ctr (*n* = 10)	-		
[84]	COS-1 to 4 (*n* = 5)	As in [83]	iPSC	NPCs, mixed glutamatergic and GABAergic forebrain neurons, Ngn2-induced excitatory neurons
	COS-6 (*n* = 4)	As in [83]		
	Ctr (*n* = 8)	As in [83]		
[85]	COS-1 to 5 (*n* = 6)	As in [83]		NPCs, mixed glutamatergic and GABAergic forebrain neurons
	COS-7 (*n* = 1)	18q22.1		
	COS-8 (*n* = 1)	8q12.3, 22q11		
	COS-9 (*n* = 1)	15q11.2, 2p25.3		
	COS-6 (*n* = 4)	As in [83]		
	COS-10 (*n* = 3)	-		
	Ctr (*n* = 10)	As in [83]		
	Ctr (*n* = 2)	-		

Abbreviations are: AOS, adult-onset schizophrenia; COS, childhood-onset schizophrenia; Ctr, control; *n*, number of case/control samples; NPC, neural progenitor cells; Ngn2, Neurogenin 2; NSD, non-spectrum disorder; Ref, reference; SAD, schizoaffective disorder; +, plus refers to new case/control samples in addition to those from the indicated reference, - refers to normal karyotype.

**Table 4:** Major differentiation methods.

Ref	Neural Induction	Patterning/Neural Progenitor Cells→Neural Cells
[73]	EB-/rosette formation	N2, WNT3A→N2, B27, BDNF, GDNF, IGF1, WNT3, cAMP
[74]	SB431542 + Dorsomorphin	N2, B27, bFGF→N2, B27, BDNF, GDNF
[75]	EB-formation + Noggin	FGF2, Shh or Wnt3a or BMP4→FGF2, EGF
[76]	EB-formation + Dorsomorphin	FGF2→N2, BDNF, GDNF, IGF1, WNT3, cAMP
[77]	As in [76]	As in [76]
[78]	As in [75]	As in [75]
[79]	SB431542 + CHIR99204	N2, B27, Dorsomorphin, RA
[80]	Rosette formation, N2, bFGF	N2, BDNF
[81]	Ngn2-mediated iN	N2, B27, BDNF, NT3→mouse glia, Ara-C
[82]	EB-/rosette formation	StemCell Induction medium™→as above
[83]	SB431542 + LDN-193189	N2, mTeSR™→BDNF, cAMP, AA→BrainPhys™
[84]	SB431542 + LDN-193189	N2, B27-RA, FGF2
	SB431542 + LDN-193189	N2, B27-RA, FGF2→B27-RA, BDNF, GDNF, cAMP, Ara-C, astrocytes, N2 B27-RA
[85]	Ngn2-mediated iN	BDNF, GDNF, cAMP, Ara-C, astrocytes
	SB431542 + LDN-193189	N2, B27-RA, FGF2→B27-RA, BDNF, GDNF, cAMP, Ara-C, astrocytes, N2

Abbreviations are: AA, ascorbic acid; Ara-C, arabinoside C; B27, B27 supplement; BDNF, brain derived neurotrophic factor; BMP, bone morphogenetic protein; cAMP, cyclic adenosine monophosphate; FGF2, fibroblast growth factor 2; EB, embryoid body; GDNF, glial cell derived neurotrophic factor; N2, N2 supplement; Ngn2, neurogenin 2; RA, retinoic acid; SHH, sonic hedgehog; Wnt, wingless.

**Table 5:** Major methods and findings on COS associated CNVs and on COS.

Ref	Major Methods	Major Findings on COS and Associated CNVs
[73]	Microarray, WCPC	Delayed decline of pluripotency markers in AOS with 22q11.2
[74]	WCPC, single cell Ca^2+^ imaging and PCR	Dysregulation of genes relevant to GABAergic, glutamatergic, and dopaminergic in electrical active neurons
[75]	Whole genome sequencing, postmortem brain	Increased L1 retrotransposition in postmortem brain from patients with AOS and iPSC derived neurons from AOS patients with 22q11.2 deletion
[76]	MicroRNA profiling	32 miRNAs are upregulated in neurons with 22q11.2 microdeletion, miRNA deregulation is broadly shared across AOS, SAD, and COS
[77]	Paired-end mRNA sequencing	Perturbed neuronal MAPK signaling, differentially expressed genes from the 22q11.2 microdeletion act during critical periods of development
[78]	miRNA and mRNA arrays	Reduced neurosphere size, neural differentiation, neurite outgrowth, cellular migration, and expression of miR-17/92 cluster and miR-106a/b that inhibit p38a (MAPK14) expression, p38 inhibitors improve diminished neurogenic-to-gliogenic ratio
[79]	ICC/IHC, complementation and knock-down experiments	Defects in adherens junctions and apical polarity. Displacement of radial glia cells leads to cortical malformation during mouse development
[80]	ICC, IB	Lower expression of CYFIP1 and PSD-95, altered dendritic morphology
[81]	Gene editing, iNeurons, electrophysiology	Reduced spontaneous mEPSC frequency, but not amplitude, and decrease in evoked EPSC amplitude. Unaltered electrical properties of human neurons, synapse numbers, and dendritic arborization
[82]	Histomorphology, electro-physiology	16p del- and 16p dup-derived NPCs show opposing differences in soma size and arborization, reduced excitability in 16p del-derived neurons, increased potassium current density in 16p dup-derived neurons, lower density of excitatory synapses in 16p del- and 16p dup-derived neurons associates with increased amplitude of mEPSCs
[83]	digital miRNA profiling	Downregulation of miR-9, a regulator of neurogenesis and of radial migration
[84]	IB, IHC, IP, knock-down	Increased STEP_61_ protein expression in forebrain neurons impairs NMDAR signaling
[85]	mRNA sequencing	Transcriptional signatures of NPCs and neurons show concordance with postmortem case/control brain samples from SCZ, BP, and ASD after adjusting for cell type composition

Abbreviations are: AOS, Adult Onset Schizophrenia; ASD, Autism Spectrum Disorder; BP, Bipolar Disorder; COS, Childhood Onset SCZ; IB, immunoblot; ICC, immunocytochemistry; IHC, immunohistochemistry; IP, immunoprecipitation; mEPSC, miniature excitatory postsynaptic current; NPC, neuronal progenitor cell; WCPC, whole cell patch clamp.
